# Pre- and post-pandemic comparisons in cardiovascular markers: a population-based study

**DOI:** 10.3389/fcvm.2024.1434141

**Published:** 2025-01-14

**Authors:** Mayssam Nehme, Maria Eugenia Zaballa, Serguei Rouzinov, Julien Lamour, Silvia Stringhini, Idris Guessous

**Affiliations:** ^1^ Division of Primary Care Medicine, Geneva University Hospitals, Geneva, Switzerland; ^2^ Faculty of Medicine, University of Geneva, Geneva, Switzerland; ^3^ Faculty of Medicine, School of Population and Public Health and Edwin S.H. Leong Centre for Healthy Aging, University of British Columbia, Vancouver, BC, Canada

**Keywords:** hypertension, cardiovacsular disease(s), diabetes mellitus, epidemiology, population-based

## Abstract

**Introduction:**

The COVID-19 pandemic, starting in 2020, raised concerns about potential long-term health impacts, including its effects on cardiovascular health and related biomarkers. This study part of the Bus Santé in Geneva, Switzerland, compared cardiovascular and metabolic profiles pre- (2016–2019) and post-pandemic (2023–2024) among individuals aged 30–75.

**Methods:**

Participants completed questionnaires and underwent a clinical visit, including a physical examination and fasting blood test to assess lipid and glycemic profiles. Linear regression was used to estimate results including mean systolic and diastolic blood pressure, cholesterol, and glycemic profiles, after adjusting for age, sex, smoking, and socioeconomic status. Quantile regression models were used to estimate median values.

**Results:**

A total of 4,558 participants were included. The study observed modest declines in mean glucose, cholesterol, HDL, and LDL levels post-pandemic, with stable blood pressure. The prevalence and treatment rates of diabetes, hypertension, and dyslipidemia remained consistent. Unawareness of these conditions was stable.

**Conclusion:**

Despite initial fears of a pandemic-induced health debt, results indicate healthy cardiovascular profiles post-pandemic, likely driven by improved lifestyle behaviors. This study highlights the importance of monitoring of cardiovascular health and suggests that lifestyle improvements may offset potential adverse pandemic effects in developed nations like Switzerland.

## Introduction

In 2020, the world witnessed a pandemic of historical proportions. One of the main concerns, in addition to the direct effects of SARS-CoV-2 infection, such as hospitalizations or post-COVID, were the potential indirect effects of the pandemic. Indeed, the pandemic was feared to incur a health debt (1) through changes in the access to care and in health behaviors, such as screening for other diseases, physical activity, diet, and sleep among others. These changes could eventually lead to long-term negative effects on health and chronic diseases. SARS-CoV-2 infection has also been recently associated with increases in cardiovascular outcomes (2), incident hypertension (3), and diabetes (4), compounded with the direct impact of post-COVID condition, likely increasing the burden of disease for millions of individuals affected by this condition (5).

Prior to the pandemic, population-based studies had shown improving trends in lifestyle behaviors (6), cholesterol profiles (7), and varying trends in hypertension awareness (8) and control (9). The coming years will reveal whether the COVID-19 pandemic has adversely impacted cardiovascular health profiles of the population or whether trends of overall healthier lifestyles have continued to improve cardiovascular health despite the pandemic's impact.

The objective of this population-based study was to evaluate pre- and post-pandemic cardiovascular health and biomarkers.

## Methods

At the Geneva University Hospitals, the Bus Santé study is an ongoing yearly population-based cross-sectional survey conducted since 1993 (10). Every year, an age- and sex-stratified random sample of about 1'000 Geneva residents is recruited through an invitation letter. Eligible individuals are identified through a standardized process based on an annual residential list provided by the local government. Subjects are first contacted via a mailed invitation letter, followed by up to 7 phone calls and 2 more letters. Those who are not reached after these attempts are replaced using the same procedure. The Bus Santé study resumed in 2023 after a hiatus between 2020 and 2023 due to the pandemic. In this study, we included participants of the Bus Santé aged 30–75, from the 2016–2019 (pre-pandemic) and the 2023–2024 (post-pandemic) yearly surveys. The Bus Santé study was approved by the Ethics Committee of Geneva (CCER 2022-01544).

Participants completed questionnaires on socioeconomic status, lifestyle and general health questions, and underwent a clinical visit with a research nurse. During this visit, a medical check-up was performed, and participants underwent a fasting blood test to assess lipid and glycemic profiles. After a 5-min rest in a seated position, participants had three systolic and diastolic blood pressure measurements, taken 30 s apart. An average of the three readings was used for systolic and diastolic blood pressure values. During the medical visit, fasting blood samples including glucose, total and high-density lipoprotein (HDL), plasma cholesterol and triglycerides are collected and then analyzed using commercially available enzymatic kits (Bayer Technicon Diagnostics, CV 1.4%, 1.2% and 1.5% for glucose, cholesterol and triglyceride respectively).

Unawareness of hypertension was defined as the absence of a diagnosis or treatment for hypertension in individuals with a blood pressure ≥140/90 mmHg. Unawareness of diabetes was defined as the absence of a diagnosis or treatment for diabetes in individuals with a fasting glucose ≥126 mg/dl. HDL was stratified by sex, as values differ between men and women.

Statistical analyses were performed using Stata 16.0 (Stata Corp, College Station, USA). Descriptive analyses were used to evaluate the prevalence of hypertension, diabetes, dyslipidemia, pre- and post-pandemic with 95% confidence interval and *p*-value considered significant at *p* < 0.05. Linear regression models were used to estimate mean systolic blood pressure, diastolic blood pressure, waist-hip ratio, fasting glucose, cholesterol, HDL and LDL levels after adjusting for age, sex, smoking, and socioeconomic status defined as education, profession and household income. Quantile regression models were used to estimate median values, with box plots and violin plots illustrating these results.

## Results

**Figure 1:**
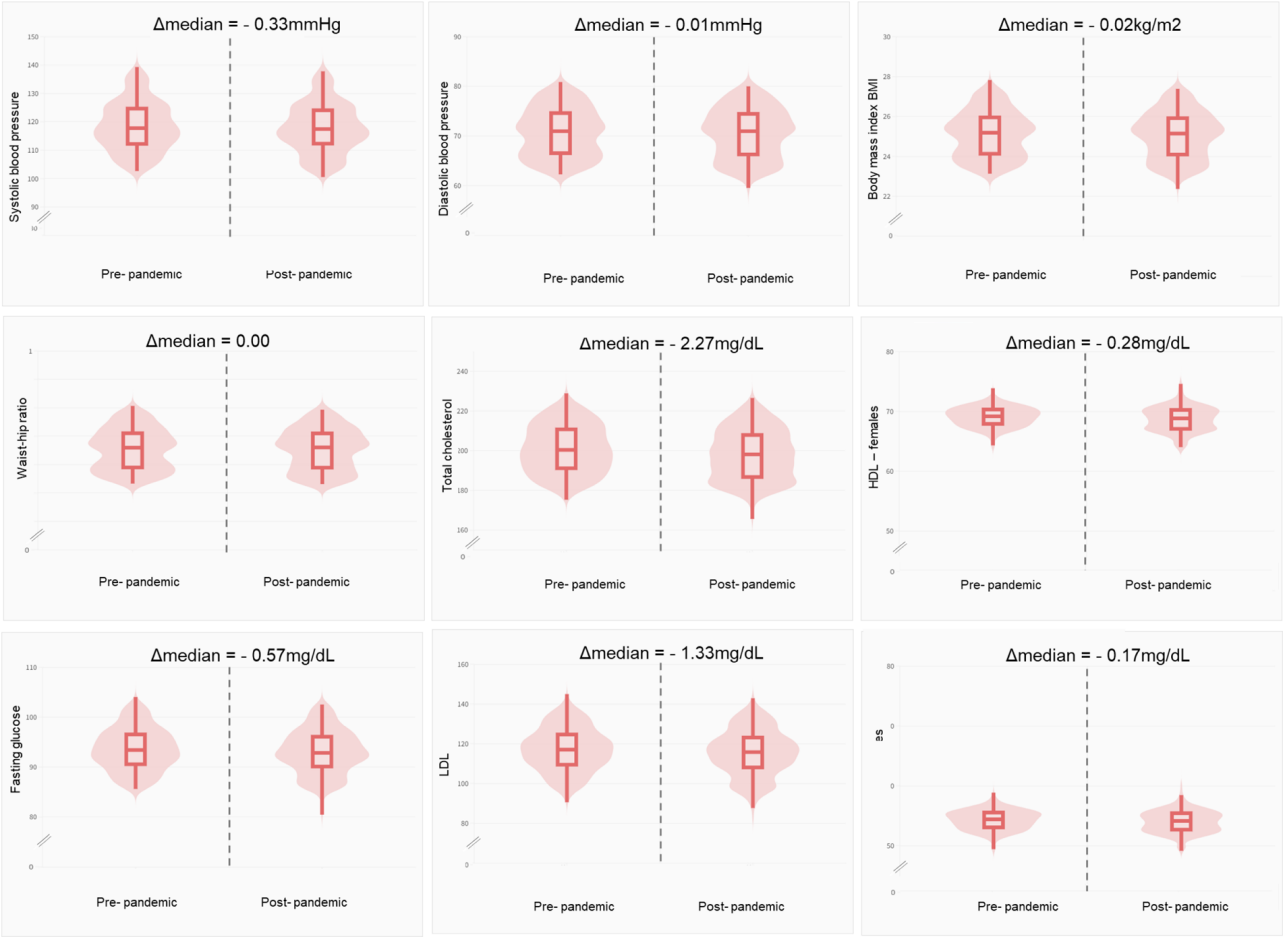
Changes in systolic and diastolic blood pressure, body-mass index, waist-hip ratio, fasting glucose and cholesterol (total, HDL, LDL) pre- (*n* = 3,849) and post-pandemic (*n* = 709). Δmedian, difference in median. Measures were adjusted for age, sex, smoking, and socioeconomic status (educational, occupational position, household income).

By February 14, 2024, *n* = 4,558 participants were included (*n* = 3,849 pre-pandemic and *n* = 709 post-pandemic). In 2023–24, 47.1% of participants were women, with a mean age of 48.2 years [standard deviation (SD) 12.1 years], 63.6% had a tertiary level of education, and 53.2% were in the high-income category as defined by the state of Geneva. In comparison, between 2016 and 2019 (pre-pandemic), 52.1% of participants were women, with a mean age of 50.2 years (SD 12.1), 55.2% had a tertiary level of education, and 40.6% were in the high-income category. The participation rate was 42% post-pandemic, compared to 55% pre-pandemic. There were no differences in body-mass index, and there was a decrease in the proportion of smokers (13.8% post-pandemic compared to 19.5% pre-pandemic) (Table 1, Figure 1).

**Table 1:** Baseline characteristics of participants pre- and post-pandemic.

	Total (*n* = 4,558)	Pre-pandemic (*N* = 3,849)	Post-pandemic (*n* = 709)	*P*-value
	*N* (%)	*N* (%)	*N* (%)	
Age (mean, SD)	49.9 (12.1)	50.2 (12.0)	48.3 (12.1)	0.001
Age groups				
30–45 years old	1,771 (38.9)	1,474 (38.3)	297 (41.9)	0.065
45–64 years old	2,116 (46.4)	1,791 (46.5)	325 (45.8)	
65 years and older	671 (14.7)	584 (15.2)	87 (12.3)	
Sex				
Female	2,340 (51.3)	2,006 (52.1)	334 (47.1)	0.061
Male	2,218 (48.7)	1,843 (47.9)	375 (52.9)	
Smoking status				
Non smoker	2,274 (50.3)	1,918 (50.1)	356 (51.2)	<0.001
Ex-smoker	1,405 (31.1)	1,162 (30.4)	243 (35.0)	
Current smoker	841 (18.6)	745 (19.5)	96 (13.8)	
Occupational position				
Salaried, low to medium skilled	2,040 (44.8)	1,779 (46.2)	261 (36.8)	<0.001
High skilled	1,063 (23.3)	886 (23)	177 (25)	
Independent	177 (3.9)	125 (3.2)	52 (7.3)	
Home maker	142 (3.1)	120 (3.1)	22 (3.1)	
Retired	611 (13.4)	521 (13.5)	90 (12.7)	
Disability-unemployed	462 (10.1)	402 (10.4)	60 (8.5)	
Did not answer - other	62 (1.4)	15 (0.4)	47 (6.6)	
Education				
Primary school	289 (6.3)	255 (6.6)	34 (4.8)	<0.001
Apprenticeship	1,053 (23.1)	917 (23.8)	136 (19.2)	
Secondary	613 (13.4)	551 (14.3)	62 (8.7)	
Tertiary	2,575 (56.5)	2,124 (55.2)	451 (63.6)	
Did not answer	8 (0.2)	0 (0)	8 (1.1)	
Other	20 (0.4)	2 (0.1)	18 (2.5)	
Household income per year				
Low	762 (17)	694 (18.1)	68 (10.8)	<0.001
Middle	1,559 (34.8)	1,361 (35.4)	198 (31.3)	
High	1,897 (42.4)	1,561 (40.6)	336 (53.2)	
Did not answer	256 (5.7)	226 (5.9)	30 (4.7)	
BMI categories				
<18.5	118 (2.7)	103 (2.8)	15 (2.2)	0.287
18.5–24.9	2,138 (49.3)	1,825 (49.9)	313 (46)	
25–29.9	1,467 (33.8)	1,221 (33.4)	246 (36.2)	
30–34.9	484 (11.2)	402 (11.0)	82 (12.1)	
35 and above	133 (3.1)	109 (3)	24 (3.5)	
Dyslipidemia	1,258 (27.6)	1,052 (27.3)	206 (29.1)	0.346
Diabetes	284 (6.2)	246 (6.4)	38 (5.4)	0.296
Hypertension	1,064 (23.3)	914 (23.7)	150 (21.2)	0.134

After adjusting for age, sex, smoking, and socioeconomic status, significant differences were found between the pre- and post-pandemic groups in mean glucose levels (Δglucose = −0.78 mg/dl, *p* < 0.001); mean cholesterol (Δchol = −2.26 mg/dl, *p* < 0.001); mean HDL (ΔHDL = −1.13 mg/dl, *p* < 0.001); and mean LDL (ΔLDL = −1.18 mg/dl, *p* < 0.001). There were no significant differences in mean systolic or diastolic blood pressure measurements between the pre- and post-pandemic periods (Table 2).

**Table 2:** Mean levels of blood pressure, glucose, cholesterol, waist-to-hip ratio and BMI pre- and post-pandemic^a^.

	Pre-pandemic	Post-pandemic	Δdifference	*P*-value
	Mean [95% CI]	Mean [95% CI]		
Overall				
Systolic blood pressure (mmHg)	119.7 [119.4–119.9]	119.0 [118.3–119.6]	−0.74	0.046
Diastolic blood pressure (mmHg)	71.6 [71.4–71.7]	71.4 [71.0–71.8]	−0.16	0.422
Fasting glucose (mg/dl)	95.3 [95.2–95.5]	95.6 [94.1–95.0]	−0.78	0.001
Total cholesterol (mg/dl)	201.8 [201.4–202.1]	199.5 [198.6–200.4]	−2.26	<0.001
HDL (mg/dl)	62.0 [61.7–62.2]	60.8 [60.3–61.4]	−1.13	<0.001
LDL (mg/dl)	118.2 [117.9–118.5]	117.0 [116.2–117.7]	−1.18	0.003
Waist-hip ratio	0.86 [0.86–0.86]	0.76 [0.85–0.86]	0.00	0.773
BMI	25.1 [25.1–25.2]	25.1 [25.0–25.2]	−0.05	0.300
Treated individuals				
Hypertension				
Systolic blood pressure (mmHg)	131.9 [131.8–132.0]	131.4 [131.2–131.7]	−0.44	0.003
Diastolic blood pressure (mmHg)	77.9 [77.8–78.0]	78.1 [77.8–78.3]	+0.15	0.301
Diabetes				
Fasting glucose (mg/dl)	135.5 [135.2–135.8]	132.7 [131.6–133.8]	−2.78	<0.001
Dyslipidemia				
Cholesterol	185.7 [185.4–185.9]	183.7 [183.1–184.3]	−1.95	<0.001
Individuals unaware				
Hypertension				
Systolic blood pressure (mmHg)	144.1 [144.0–144.2]	143.1 [142.6–143.5]	−1.00	<0.001
Diastolic blood pressure (mmHg)	94.0 [94.0–94.0]	93.1 [92.7–93.5]	−0.90	<0.001
Diabetes				
Fasting glucose (mg/dl)	141.7 [141.5–141.9]	141.7 [141.2–142.1]	0.00	0.986
Dyslipidemia				
Cholesterol	271.6 [271.6–271.7]	271.4 [271.2–271.6]	−0.24	0.002

^a^ Adjusted for age, sex, smoking, and socioeconomic status (educational, occupational position, household income).

The proportions of individuals with diabetes, hypertension, or dyslipidemia did not differ between the pre- and post-pandemic periods, nor did the proportions of individuals receiving treatment for these conditions. Around 53.5% of individuals with diabetes, 25.9% of individuals with dyslipidemia and 47.9% of individuals with hypertension were receiving treatment. In a subgroup analysis of treated individuals, there were no significant differences in blood pressure, glycemic or cholesterol profiles pre- and post-pandemic. The risk of cardiovascular disease including cardiac infarction, angina pectoris, PAD, stroke was 5.6% in our study population.

Among individuals with a blood pressure ≥140/90 mmHg, 4.4% [4.3–4.6] were unaware of having hypertension pre-pandemic, and this remained similar post-pandemic at 4.4% [4.1–4.5]. Among individuals with a fasting glucose ≥126 mg/dl, 1.3% [1.2–1.3] were unaware of having diabetes in the pre-pandemic group, and this remained similar post-pandemic at 1.1% [1.0–1.2]. Hypertension and diabetes unawareness were significantly higher in men and individuals aged 45–64 but did not differ by socioeconomic status. In a subgroup analysis of individuals unaware of having hypertension or dyslipidemia, post-pandemic trends showed a decrease in blood pressure and cholesterol: mean systolic blood pressure (Δsystolic blood pressure = −1.00 mmHg, *p* < 0.001); mean diastolic blood pressure (Δdiastolic blood pressure = −1.00 mmHg, *p* < 0.001) and mean cholesterol (Δchol = −0.24 mg/dl, *p* = 0.002); with no differences in glucose levels.

## Discussion

Our results showed unchanged to modest decreases in glycemic and cholesterol profiles with stable blood pressure measurements when comparing pre- and post-pandemic individuals in the same source population. Individuals in the post-pandemic period appeared overall healthier, with trends toward lower smoking rates and improved cardiovascular markers even after adjusting for age, sex and socioeconomic status.

While studies early in the pandemic studies showed potential long-term negative effects on chronic diseases (1), this study is one of the first post-pandemic studies to use objective data comparing pre- and post-pandemic blood pressure, cholesterol and glycemic profiles, even in individuals unaware of having a condition. Unawareness, treatment levels, and the prevalence of diabetes, hypertension, and dyslipidemia remained unchanged in pre- and post-pandemic periods. In individuals unaware of hypertension or high cholesterol, measurements were lower post-pandemic compared to pre-pandemic. Similarly, treated individuals had lower systolic blood pressure, glycemia and cholesterol levels post-pandemic compared to pre-pandemic.

This study further supports the hypothesis that individuals in developed countries such as Switzerland, may be following healthier lifestyle behaviors, resulting in better cardiovascular and glycemic control, with values trending lower in the general population. Previous results from the same source population (Bus Santé), showed improving trends in cardiovascular risk factors in the years leading up to the COVID-19 pandemic (10); and this current study indicates that these trends may have continued even after the pandemic. However, it is still important to consider the direct effects of SARS-CoV-2 infection on morbidity and mortality, with a sharp increase in cardiovascular and overall mortality especially early in the pandemic (11), followed by a decline, and the long-term effects especially in individuals with post-COVID condition (5). Ongoing research will reveal whether the incidence of chronic diseases evolves and potential links to SARS-CoV-2 infection.

Limitations include potential differences in the baseline characteristics between the pre- and post-pandemic groups. However, adjusting for age, sex, smoking and socioeconomic factors likely addressed this issue. Potentially having more mortality during COVID-19 in vulnerable groups could have artificially reduced the difference of main diseases and risk factors pre- and post-pandemic The difference in population size between the pre- and post-pandemic study groups likely affects power as well, and differences in results should be carefully interpreted. Standardization to the general population showed similar results, suggesting potential generalizability. We lack information on SARS-CoV-2 infection status, though most participants were likely exposed to the virus by 2023. Additionally, we lack information on post-COVID condition which could have contributed to chronic diseases including diabetes (12) and cardiovascular disease (2).

This study suggests that the impact of COVID-19 on cardiovascular health may be less severe than initially predicted during the pandemic, potentially mitigated by healthier lifestyle behaviors. The use of new medications such as GLP-1 analogs, SGLT-2 inhibitors for diabetes and medications for dyslipidemia has been increasing with time and should be accounted for in the control of disease, in addition to lifestyle behaviors. Continued screening and follow-up of hypertension, diabetes and dyslipidemia should remain *a priori*ty. Larger and more extended studies are necessary to ensure the reliability and validity of the results, and to assess the long-impact of COVID-19 on health.

## Data Availability

The raw data supporting the conclusions of this article will be made available by the authors, without undue reservation.
